# Prolactin level changes according to atypical antipsychotics use

**DOI:** 10.1192/j.eurpsy.2025.2079

**Published:** 2025-08-26

**Authors:** J. Hong, ?.-H. Jeong

**Affiliations:** 1 Psychiatry, Iksan hospital, Iksan; 2 Psychiatry, St. Vincent’s Hospital, Suwon, Korea, Republic Of

## Abstract

**Introduction:**

Antipsychotic drugs are known as the major cause of non-neoplastic hyperprolactinemia.

**Objectives:**

This study aimed to investigate the levels of serum prolactin elevation depending on the use of antipsychotic drugs in patients through the Clinical Data Warehouse.(CDW)

**Methods:**

We conducted a cohort search in the CDW application with the following condition. The subjects who aged more than 18 years old and visited from March 1, 2017, to May 31, 2022 at the Department of St. Vincent’s Hospital, The Catholic University of Korea. The subjects were diagnosed according to ICD-10 for schizophrenia, schizotypal and delusional disorders, manic episodes, and bipolar affective disorders. And the subjects who was taking one of risperidone, blonanserin, amisulpride, and olanzapine. The subjects also conducted serum prolactin level tests. After that, we reviewed the medical data.

**Results:**

Among the 117 subjects included in the analysis, the mean serum prolactin level was 64.654.6ng/ml. Serum prolactin levels were significantly higher in subjects taking risperidone or amisulpride compared to blonanserin and olanzapine. The female subjects who took blonanserin, olanzapine, and risperidone had significantly higher prolactin levels, but there was no difference in serum prolactin levels between the sex in the subjects who took amisulpride.

**Conclusions:**

This study suggests the need for regular monitoring of serum prolactin levels in patients who are taking antipsychotics, especially in female patients. And we showed that there is a possibility to conduct more effective and simpler big data research using the CDW. Further studies on the subjects with controlled confounding variables and larger sample groups are needed.

**Disclosure of Interest:**

None Declared

